# Alkyl-*π* engineering in state control toward versatile optoelectronic soft materials

**DOI:** 10.1088/1468-6996/16/1/014805

**Published:** 2015-02-25

**Authors:** Fengniu Lu, Takashi Nakanishi

**Affiliations:** 1 International Center for Materials Nanoarchitectonics (MANA), National Institute for Materials Science (NIMS) 1-2-1 Sengen, Tsukuba 305-0047, Japan; 2 Warsaw University of Technology, Warsaw 02-507, Poland; 3 Institute for Molecular Science (IMS), 5-1 Higashiyama, Myodaiji, Okazaki 444-8787, Japan

**Keywords:** self-assembly, *π*-conjugated molecules, alkyl chains, optoelectronic, liquid

## Abstract

Organic *π*-conjugated molecules with extremely rich and tailorable electronic and optical properties are frequently utilized for the fabrication of optoelectronic devices. To achieve high solubility for facile solution processing and desirable softness for flexible device fabrication, the rigid *π* units were in most cases attached by alkyl chains through chemical modification. Considerable numbers of alkylated-*π* molecular systems with versatile applications have been reported. However, a profound understanding of the molecular state control through proper alkyl chain substitution is still highly demanded because effective applications of these molecules are closely related to their physical states. To explore the underlying rule, we review a large number of alkylated-*π* molecules with emphasis on the interplay of van der Waals interactions (vdW) of the alkyl chains and *π*–*π* interactions of the *π* moieties. Based on our comprehensive investigations of the two interactions’ impacts on the physical states of the molecules, a clear guidance for state control by alkyl-*π* engineering is proposed. Specifically, either with proper alkyl chain substitution or favorable additives, the vdW and *π*–*π* interactions can be adjusted, resulting in modulation of the physical states and optoelectronic properties of the molecules. We believe the strategy summarized here will significantly benefit the alkyl-*π* chemistry toward wide-spread applications in optoelectronic devices.

## 1. Introduction

The last few decades have witnessed the prosperous development of optoelectronic devices based on organic/polymer materials, such as light-emitting diodes [1–3], photovoltaic devices [1, 4], field-effect transistors [5–7] and electronic devices [8, 9], by virtue of their extensive well-known and potential applications. Compared with their inorganic counterparts, these organic ones are much more advantageous in view of their light weight, low cost, flexibility, unlimited selection of building blocks and convenience for large area fabrications [10].

In general, *π*-conjugated molecules are intriguing building motifs for the design and construction of organic optoelectronic devices on account of their rich and desirable electronic and optical properties [11, 12]. Moreover, the optoelectronic characteristics can be facilely tailored through chemical functionalization of the molecules. As a result, extensive organic *π*-conjugated molecules with a wide range of structures have been synthesized and fabricated into various optoelectronic devices. However, most *π*-conjugated molecules, restricted by the strong *π*–*π* interactions among the *π*-conjugated moieties, exist as solids at room temperature. These solid materials, either amorphous or crystalline, suffer poor processability for the application of flexible optoelectronic devices. In addition, large *π*-conjugated systems have a strong tendency to form random stacks and aggregates with extremely poor solubility in organic solvents. This limits the handling of the molecules in conventional solution-process techniques [13, 14] such as spin-coating and printing for device fabrications. As another issue, *π*-conjugated systems can often decompose upon oxygen attack and undergo dimerization or polymerization upon exposure to external stimuli.

**Scheme 1.**
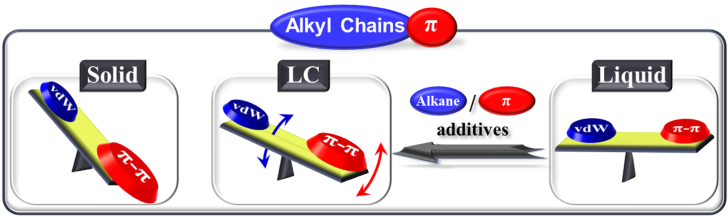
State control of alkyl-*π* molecules by adjusting van der Waals (vdW) and *π*–*π* interactions through proper alkyl chain substitution or the introduction of additives.

The most frequently used method to overcome these issues is to attach solubilizing alkyl chain groups to the *π*-conjugated moieties [15, 16], which can not only soften the *π*-conjugated materials to some extent but also enhance the solubility and protect the *π* units from oxygen attack and external stimuli. Fundamentally, upon the appending of alkyl chains, van der Waals (vdW) interactions (vdW interactions mentioned in this review are specific for the vdW interactions of alkyl chains) of the chains were introduced, which can interplay with the *π*–*π* interactions of the *π* moieties. Based on a comprehensive investigation of various alkylated-*π* molecular systems reported by our group and other research teams, we find that the physical states of the molecules can be dominated by simply tuning the balance of vdW and *π*–*π* interactions (scheme 1). When the vdW interactions are far weaker than the *π*–*π* interactions, the alkyl-*π* molecules are in a solid state. These molecules, assisted by solvents, can assemble into various structures. With increasing vdW interactions and periodic segregation of the rigid *π*-conjugated moieties by the alkyl chains, thermotropic liquid crystals (LCs) can be produced. Further increasing of the vdW interactions may result in a delicate balance with the *π*–*π* interactions, which would generate a solvent-free liquid state in which both alkyl chains and *π*-moieties are disordered.

Because of the strong effect of alkyl chains on vdW interactions, the balance of the vdW and *π*–*π* interactions can be adjusted by diversifying the alkyl chains or modulating the chain substitution pattern. Moreover, we demonstrated that the introduction of alkane or *π* additives can break the balance between the vdW and *π*–*π* interactions in liquid molecules, allowing additive-directed conversion from a liquid to a highly ordered LC or a gel [17]. Previously, our group had reviewed assembled functional materials with a focus on the smart combination of *π* molecules and alkyl chains [18–22]. In contrast, herein, we stress ‘state control’ through alkyl-*π* engineering based on a deep investigation of a large number of alkylated-*π* molecules. We aim to provide clear guidance for mastering the balance between vdW and *π*–*π* interactions in alkylated-*π* molecules and to direct their physical states and state-dependent optoelectronic applications.

## 2. Solvent-assisted solid self-assemblies of linear alkyl chain-attached *π* molecules

The majority of *π* molecules with attached linear alkyl chains appear to be solid at room temperature. Because optoelectronic device performance is strongly dependent on the precise organization of the *π*-conjugated moieties [23], controllable self-assembly of these solid state molecules is required for achieving excellent optoelectronic properties [24, 25]. The self-assembly behavior of these alkylated-*π* molecules is intrinsically affected by the alkyl chain substitution pattern due to the strong interplay of the alkyl chains’ vdW interactions with the *π* units’ *π*–*π* interactions. Moreover, within solvent systems, adjustment of external experimental conditions (solvent polarity and temperature), as well as the introduction of substrates and other interactions (electrostatic, hydrogen bonding and hydrophilic interactions), can all play significant roles in forming numerous self-assembled nano/micro structures.

### 2.1. Solvent polarity-modulated architectures

**Figure 1.**
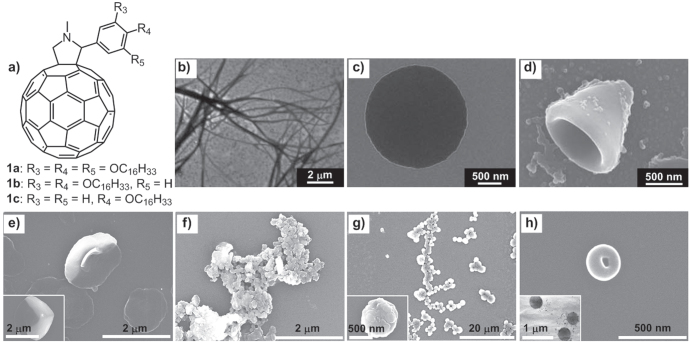
(a) Chemical structures of C_60_ derivatives **1a**–**1c** containing linear alkyl chains. Scanning electron microscopy (SEM) images of fibrous structures of **1a** assembled from 1-propanol (b), a nanodisk of **1a** formed from 1,4-dioxane (c) and a conical object of **1a** assembled from a 1:1 tetrahydrofuran (THF)/H_2_O mixture (d). Reprinted from [26]. SEM images of disk-like sheets of **1b** formed from a 2:1 2-propanol/toluene mixture (e), SEM images of self-aggregated particles of **1b** obtained from a 1:2 THF/H_2_O mixture (f), globular aggregates of **1(c)** with coarse surfaces formed from a 2:1 2-propanol/toluene mixture (g) and SEM and transmission electron microscopy (TEM) (inset) images of vesicular-spherical objects of **1c** assembled from a 1:2 THF/H_2_O mixture (h). Parts (e)–(h) reprinted from T Nakanishi *et al* 2008 *Thin Solid Films* **516** 2401, © 2008 with permission from Elsevier.

Our group has reported a series of linear alkyl chain-attached C_60_ derivatives **1a**–**1c** (figure 1(a)), which self-assembled into diverse well-defined 1D, 2D and 3D architectures in different organic solvents. The self-assembly of **1a** appended with 3,4,5-trishexadecyloxyl chains, prepared simply by cooling a solvent mixture from 60 °C to 20 °C, gave rise to a variety of self-assembled architectures under different solvent conditions. 1D nanofibers (figure 1(b)), 2D nanodisks (figure 1(c)) and 3D cones (figure 1(d)) were obtained in 1-propanol, 1,4-dioxane and a 1:1 tetrahydrofuran (THF)/H_2_O mixture, respectively [26]. Similarly, with identical preparation procedures, 3,4-bishexadecyloxyl chains attached **1b** formed 2D disk-like sheets in a 2:1 2-propanol/toluene mixture (figure 1(e)) and rather random 3D self-aggregated particles in a 1:2 THF/H_2_O mixture (figure 1(f)). The 4-hexadecyloxyl chain modified **1c** created 3D globular aggregates in a 2:1 2-propanol/toluene mixture (figure 1(g)) and 3D vesicular-spherical objects in 1:2 THF/H_2_O mixtures (figure 1(h)) [27].

Such solvent-dependent self-organization originates from the amphiphilicity of the two components, C_60_ and alkyl chains, in organic solvent. Although both are hydrophobic, the sp^2^-carbon rich C_60_ moiety shows higher solubility in aromatic solvents, such as toluene, while the sp^3^-carbon rich alkyl chains exhibit stronger affinity to aliphatic alkanes and polar solvents such as alcohols and ethers. With these amphiphilic-like features, the two moieties go through different solvation in these solvent systems, which would remarkably influence the curvature of the ensuing assembly and result in various self-assembled structures in the nano and micrometer scales. Fine-tuning of the morphologies of these assembled objects by simply adjusting the solvent systems can generate extensive hierarchical assemblies and strikingly enrich the structures and functions of those materials.

**Figure 2.**
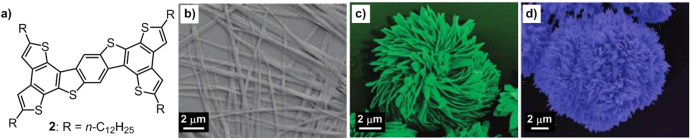
(a) Chemical structure of oligoarene derivative **2** containing linear alkyl chains. False-color SEM images of different nanostructures of **2**: microbelts self-assembled from 1,4-dioxane (b), flower-A formed from THF (c) and flower-B assembled from *n*-decane (d). Reprinted with permission from L Wang *et al* 2009 *Langmuir* **25** 1306, © 2009 American Chemical Society.

Wang *et al* reported a linear dodecyl chain-substituted oligoarene derivative, **2**, which exhibited similar solvent-dependent self-assembly behavior as **1a**–**1c** (figure 2(a)) [28]. By drop casting solutions of **2** in different solvents onto glass substrates, three distinctive structures were obtained after evaporation of the solvents. In 1,4-dioxane, **2** self-assembled into 1D microbelts on the order of tens of micrometers in length, several hundred nanometers in width and 50 nm in thickness (figure 2(b)). However, in THF and *n*-decane, two different 3D flower-shaped microstructures, flower-A (figure 2(c)) and flower-B (figure 2(d)), were generated. In spite of their similar diameters, around 10–20 *μ*m, flower-A was made of hundreds of shuttle-like 1D petals, while flower-B was composed of hundreds of 1D acicula-like petals. In the light of their high surface areas, the flower-shaped objects were fabricated for explosive detection because the detection scope mainly relied on surface area. The detection speed of 3D flower-B was enhanced by more than 700 times compared with that of the 1D microbelts, providing prospects for using these self-assembled structures in chemosensing.

### 2.2. Temperature-influenced architectures

**Figure 3.**
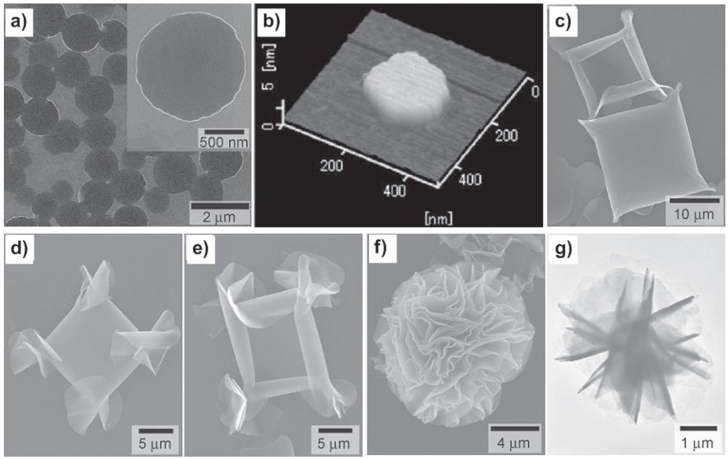
SEM (a) and atomic force microscopy (AFM) (b) images of nanodisks formed by cooling a 1,4-dioxane solution of **1a** from 60 °C to 20 °C. SEM (c) image of square-shaped objects loosely rolled up in each corner formed by rapid cooling of 1,4-dioxane solution of **1a** from 60 °C to 5 °C. SEM images (d), (e) of the further rolled up objects of crumpled structures at the four corners. SEM (f) and TEM (g) images of final flower-shaped assemblies of **1a** precipitated by slow aging at 5 °C. Reprinted with permission from T Nakanishi *et al* 2007 *Small* **3** 2019, © 2007 John Wiley & Sons.

In addition to the solvent effect, temperature also plays a significant role in the self-assembly behaviors. Upon heating at 60 °C, the mixture of **1a** in 1,4-dioxane (1.0 mM) transformed into a transparent light-brown solution, which formed aggregates composed of nanodisks after subsequent aging at 20 °C for 24 h (figure 3(a)). The nanodisks have 0.2–1.5 *μ*m diameters and a thickness of 4.4 nm, which is in good agreement with the thickness of the alkyl chain-interdigitated bilayers (figure 3(b)) [26]. Interestingly, further cooling of the mixture from 20 °C to 5 °C and keeping at 5 °C for 12 h resulted in precipitates comprising flower-shaped assemblies several micrometers in size (3–10 *μ*m) with crumpled sheet- or flake-like nanostructures several tens of nanometers in thickness (figures 3(f)–(g)) [29].

Such temperature-dependent morphologies have significantly promoted the understanding of the formation mechanism of the very complex flower-shaped objects. Given that bending of a thin sheet is entropically more favorable than the stretched state, the pre-formed disk objects at 20 °C have a strong tendency to roll up at the edges (figure 3(c)), which are attainable upon rapid cooling of the solution from 60 °C to 5 °C. The continual rolling up would result in spatial congestions at the four corners, leading to crumpling, bending, stretching and fracture of the disks (figures 3(d)–(e)). Once these conformations complete, the bilayer at the edges keep on growing to fix the spatial conformation of the crumpled sheets, resulting in the flower-shaped superstructures (figures 3(f)–(g)). According to this process, the slow temperature aging is indispensable for the growth from small nanodisks to microscopic flower-shaped objects. This result reveals the considerable influence of temperature on the self-organization process.

**Figure 4.**
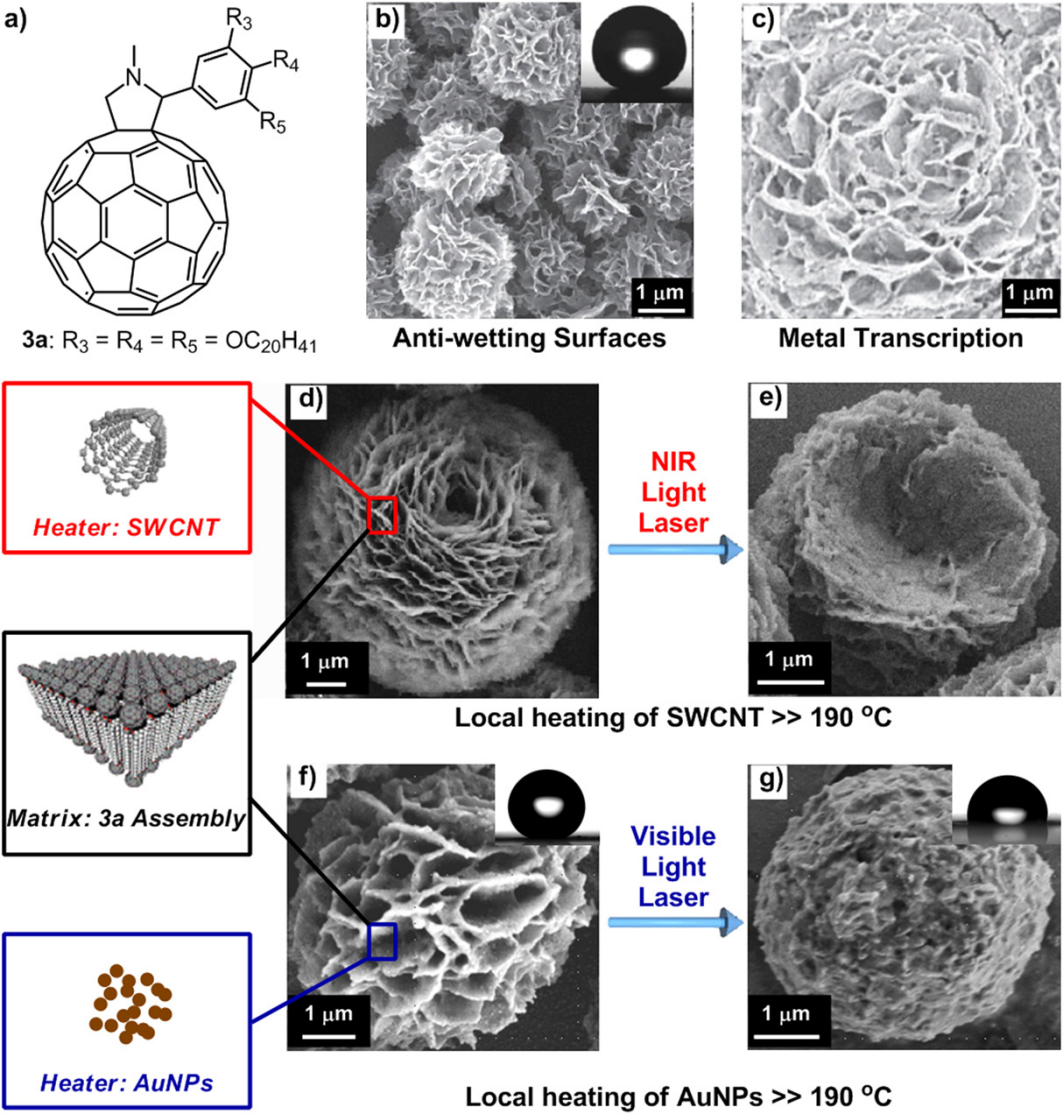
(a) Chemical structure of an alkylated-C_60_ **3a**. (b) SEM image of globular microparticles with nanoflaked outer surfaces formed by cooling a 1,4-dioxane solution of **3a** from 70 °C to 20 °C and a photograph (inset) of a water droplet on the surface of a thin film of the globular micro-objects on a Si substrate. Reprinted with permission from T Nakanishi *et al* 2008 *Adv. Mater.* **20** 443, © 2008 John Wiley & Sons. (c) SEM image of Au nanoflakes transcribed from the nanoflake-featured microparticles of **3a**. Reprinted with permission from Y Shen *et al* 2009 *Chem. Eur. J.* **15** 2763, © 2009 John Wiley & Sons. Morphology of **3a**-SWCNT assembly before (d) and after (e) NIR light laser irradiation (*λ* = 830 nm). Reprinted with permission from Y Shen *et al* 2010 *J. Am. Chem. Soc.* **132** 8566, © 2010 American Chemical Society. Morphology and anti-wettability of **3a**-AuNPs before (f) and after (g) visible light laser irradiation (*λ* = 532 nm). Reprinted with permission from H Asanuma *et al* 2013 *Langmuir* **129** 7464, © 2013 American Chemical Society.

Similar self-assembly phenomenon using a heating/cooling process in 1,4-dioxane was observed for an alkylated C_60_ derivative, **3a**, bearing 3,4,5-triseicosyloxyl chains (figure 4(a)), which also formed globular objects with wrinkled nanoflake structures at the outer surface (figure 4(b)) [30]. The extraordinarily high roughness, together with the hydrophobic properties of both the C_60_ and alkyl chains, made the fabricated thin film of these globular microparticles exhibit superhydrophobicity with a water contact angle of 152° (figure 4(b), inset). The surface morphology and superhydrophobicity of the thin film is reminiscent of the self-cleaning features of the Lotus leaves.

In addition, the microparticles can also be employed as a template for nanoflaked metal surfaces by simply sputtering the desired metal directly onto a thin film of the globular objects and rinsing out of **3a** in a good solvent such as chloroform (CHCl_3_) (figure 4(c)) [31]. The resulting Au nanoflake surfaces, retaining the high roughness features, are able to fabricate both superhydrophobic and superhydrophilic surfaces through chemical modification of hydrophobic and hydrophilic thiol molecules. The Au nanoflake surfaces can also be applied as a surface-enhanced Raman scattering (SERS) active substrate owing to the plasmonic effect of nanostructured metal [32].

By virtue of the highly photothermally active single-walled carbon nanotubes (SWCNTs) [33, 34] and gold nanoparticles (AuNPs) [35], the nanoflake-featured microparticles of **3a** doped with SWCNTs or AuNPs were either employed as a temperature indicator in air [36] or applied to modulate surface anti-wetting characteristics [37]. Specifically, NIR laser-induced heating of SWCNTs could generate increased temperatures, which, once reaching the melting point of the **3a**-SWCNTs assembly (190 °C), would induce morphology changes observable by various microscopy techniques (figures 4(d)–(e)). Similarly, the surface roughness of the fabricated thin films prepared with **3a**-SWCNTs or **3a**-AuNPs could be remotely controlled by NIR light laser (*λ* = 830 nm) or visible light laser (*λ* = 532 nm) irradiation, exerting significant influence on the surface-roughness-dependent anti-wetting properties (figures 4(f)–(g)).

**Figure 5.**
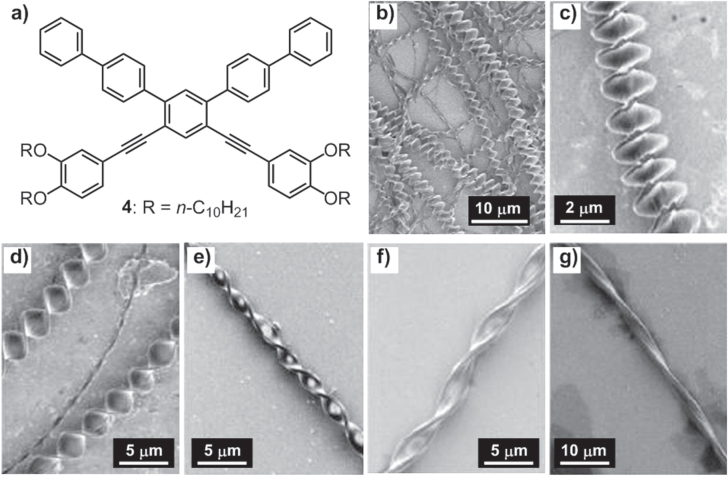
(a) Chemical structure of molecule **4**. (b) SEM image of self-assembled microwires from a solution of **4** in a 2:3 CHCl_3_/ethanol (EtOH) mixture by evaporating the solvents slowly. SEM images of self-assembled **4** precipitated by cooling of its hot homogeneous solution at different temperatures: 15 °C (c), 20 °C (d), 25 °C (e), 30 °C (f) and 35 °C (g). Reprinted with permission from H-B Chen *et al* 2009 *Langmuir* **25** 5459, © 2009 American Chemical Society.

With the same temperature-regulating self-assembly strategy, the Pei group also obtained 3D flower-shaped micro-objects using a benzothiophene derivative appended with *n*-dodecyl chains [38]. In addition, the same group reported the morphology tuning of chiral microtwists through temperature control [39]. By slowly evaporating a solution of an achiral compound **4** (figure 5(a)) in a 2:3 CHCl_3_/ethanol (EtOH) mixture (1 mg mL^−1^), perfectly twisted chiral structures with uniform pitch were obtained (figure 5(b)). Interestingly, with different precipitation temperatures, the pitch of the microtwist could be easily tuned (figures 5(c)–(g)). Basically, a higher temperature led to a slower precipitation process and thus a larger pitch. This phenomenon was explained by special crystal growth kinetics, according to which the driving force for twisting derived from the imbalance of the growth rate between the center and the edge of the self-assembled nanobelts.

### 2.3. Chain-substitution pattern-controlled architectures

**Figure 6.**
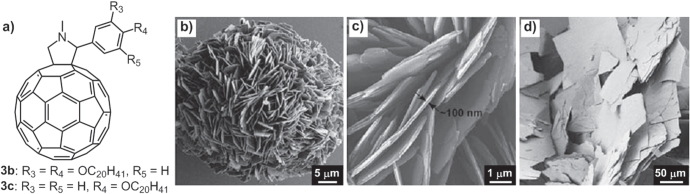
(a) Chemical structure of C_60_ derivatives **3b**–**3c**. SEM images of the assemblies of **3b**–**3c** formed by cooling their 1,4-dioxane solution from 70 °C to 20 °C (b); (c) Plate-Rich giant particles of **3b**; (d) sheet structures of **3c**. Reprinted from [40].

In addition to the solvent effect on the self-assembly of **1a**–**1c**, which was briefly described in figure 1, the chain substitution pattern also had significant influence on the control of organized architectures. Through the same self-assembly procedures and same temperature history within the same solvent, alkylated-C_60_ derivatives, **3a**–**3c**, bearing different eicosyloxy chain numbers (figures 4(a), 6(a)), formed different self-assembled structures [40]. Specifically, compound **3a** appended with 3,4,5-triseicosyloxyl chains formed globular microparticles with a nanoflaked outer surface (figure 4(b)). While **3b** bearing 3,4-biseicosyloxyl chains generated plate-rich giant particles (figures 6(b)–(c)). The 4-monoeicosyloxyl chain-substituted **3c**, on the other hand, gave rise to sheet structures (figure 6(d)). The distinctive morphologies can be attributed to the competing *π*–*π* interactions of neighboring C_60_ moieties with the vdW interactions of the alkyl chains. With fewer alkyl chains, the *π*–*π* interaction of C_60_ moeties is richer and therefore induces plate-rich architectures due to the lower flexibility of the molecular organizations. On the other hand, with more alkyl chains, the *π*–*π* interaction of C_60_ moieties is constrained, resulting in a suppressed planar arrangement of C_60_ moeties and thus favoring globular objects with wrinkled nanoflaked outer surfaces.

### 2.4. Substrate-supported self-assemblies

**Figure 7.**
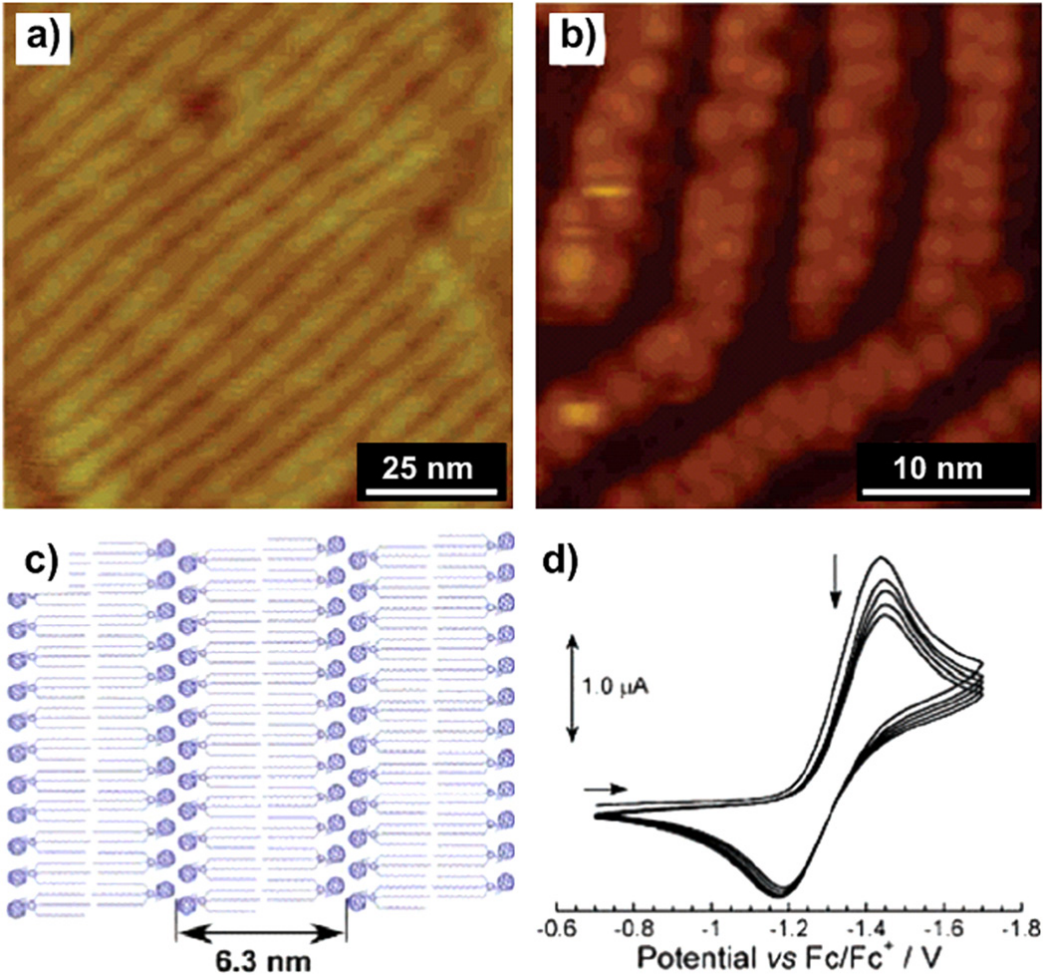
AFM (a) and high-resolution scanning tunneling microscopy (STM) (b) images of **1a** on HOPG spin-coated from a CHCl_3_ solution. (c) Schematic illustration showing the molecular organization of **1a** in the lamellae. (d) Cyclic voltammogram of **3a** on HOPG (0.1 M tetra-*n*-butylammonium perchlorate, acetonitrile (CH_3_CN), Ar atmosphere, scan rate 0.1 V s^−1^, 20 °C). Reprinted with permission from T Nakanishi *et al* 2006 *J. Am. Chem. Soc.* **128** 6328, © 2006 American Chemical Society.

The interaction between the appended alkyl chains of alkylated-*π* molecules and solid substrates, highly oriented pyrolytic graphite (HOPG) in particular, was reported to be able to drive the corresponding *π* molecules to form lamellae and other complicated ordered structures and therefore organize into epitaxially ordered molecular patterns [41, 42]. This technique, although widely utilized for the establishment of 2D alignments [43, 44], was seldom employed for the construction of 1D architectures [45]. Taking into consideration the significance of the 1D C_60_ structure in electronic nanodevices, our group applied the technique to fabricate perfectly aligned 1D C_60_ nanowires by spin-coating a dilute CHCl_3_ solution of compound **1a** onto HOPG surfaces (figure 7(a)) [46]. The nanowires, with the C_60_ heads locating at the center in a zigzag fashion and the substituted alkyl chains stretching outward (figure 7(b)), possess lengths exceeding several hundred of nanometers. The periodicity of the nanostripes corresponds well to twice the molecular length of **1a**, revealing a perfect lamellar structure with fully extended alkyl chains of all-trans conformation (figure 7(c)).

The perfect alignment is mainly driven by the good lattice matching between the all-trans conformational alkyl chains and graphite, which force the alkyl chains to assemble along the underlying lattice axis of the basal plane of graphite. Meanwhile, the *π*–*π* interactions allow the C_60_ units to form in a zigzag fashion on the surface. This hypothesis was further supported by the similar alignment behavior of **1b** and **3a** [47].

Notably, even in the surface-confined assemblies, the C_60_ groups, i.e. **3a**, showed fully maintained electrochemical activity (figure 7(d)), suggesting these molecules as promising candidates for electronic nanodevices. Moreover, such 1D nanowires could facilely regulate the carrier transporting direction, which could be of prominent advantage for applications in semiconductors [48–51].

**Figure 8.**
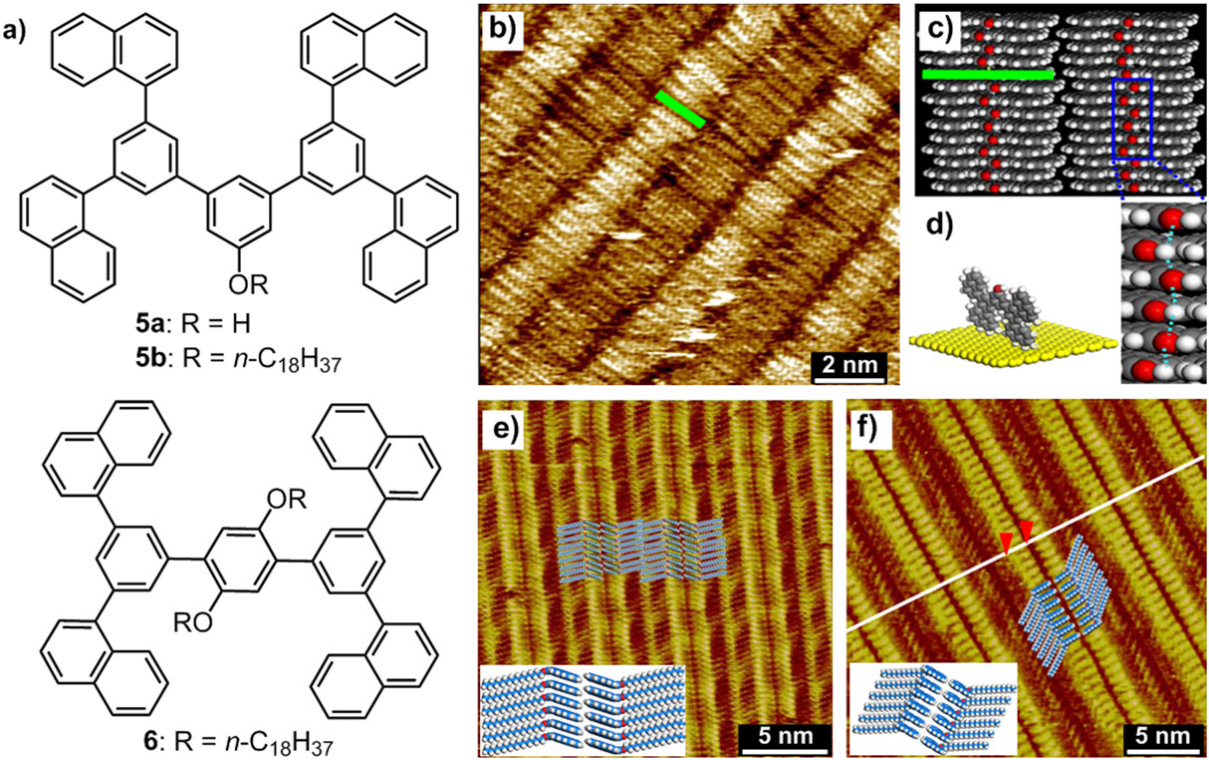
(a) Chemical structures of dendronized conjugated molecules **5** and **6**. (b) High-resolution STM image of the **5a** monolayer on the HOPG surface. (c) Schematic model of the **5a** monolayer adopting an edge-on stacking pattern, and an enlarged structural model showing hydrogen bonds by blue dashed lines. (d) Side-view model of an individual **5a** molecule on the HOPG surface. (e) High-resolution STM image of **5b** on the HOPG surface; inset, proposed structural model of the **5b** adlayer. (f) High-resolution STM image of the self-assembly of **6** on the HOPG surface; inset, a possible packing pattern of the molecular lamellar structure of **6**. Reprinted from Y Yang *et al* 2012 *Appl. Surf. Sci.* **263** 73, © 2012 with permission from Elsevier.

In addition, the aligning strategy on the substrate surface constructed here has been generalized to other functional molecules. The Miao group have investigated a series of dendronized molecules **5a**, **5b** and **6** (figure 8(a)), which formed 2D self-organized monolayers on the HOPG surface through solution evaporation of the molecules under ambient conditions [52]. As a result of the *π*–*π* interactions, the dendronized conjugated moieties of all the compounds adopted an edge-on arrangement on the HOPG surface. Substituted by a hydroxyl group, molecule **5a** stood perpendicularly to the substrate surface, resulting from both intermolecular *π*–*π* interactions and hydrogen bonding (figures 8(b)–(d)). Molecule **5b**, however, attached by one long alkyl chain, was subjected not only to the *π*–*π* interactions but also to the vdW interactions of alkyl chains, as well as the interactions between the alkyl chains and the HOPG substrate. As a consequence, the alkyl chains laid flat on the substrate, while the conjugated units stood perpendicular to the HOPG surface (figure 8(e)). Compound **6** exhibited a similar 2D adsorbed structure to that of **5b**, except for the slightly larger intermolecular spacing, which was due to the tilted conjugated units from the HOPG surface (figure 8(f)). In addition, both **5b** and **6** displayed zigzag carbon skeletons of the alkane molecules relative to the HOPG substrate, which was due to a subtle interplay of packing and entropic effects [53].

Apart from this example, Chen *et al* have also reported a 2D self-assembly, which formed on the HOPG substrate with a long alkyl chain-substituted oligo(*p*-phenylene vinylene) (OPV) derivative [54]. Mali *et al* described a concentration-controlled 2D structural evolution of a large triangular discotic macrocycle containing an alkyl chain at the 1,2,4-trichlorobenzene/HOPG interface [55]. Recently, Xu *et al* [56] and [57] Tamaki *et al* independently reported the odd–even (alkyl chain carbon number) effect on the self-assembly structures of fluorenone and anthraquinone derivatives, respectively, on HOPG. More strikingly, a 3D nanowire based ‘organic radical’ unit on the HOPG surface, formed from a polychlorotriphenyl radical bearing three long alkyl chains, was also reported [58].

### 2.5. Other interaction-assisted self-assemblies

With proper molecular design, other interactions, such as electrostatic, hydrogen bonding or hydrophilic interaction, can be introduced to alkylated-*π* molecules. Any of these interactions, interplaying with the *π*–*π* interaction of the *π* moieties and the vdW interaction of the alkyl chains, would develop versatile self-assembled structures with extensive functions.

#### 2.5.1. Electrostatic interaction

**Figure 9.**
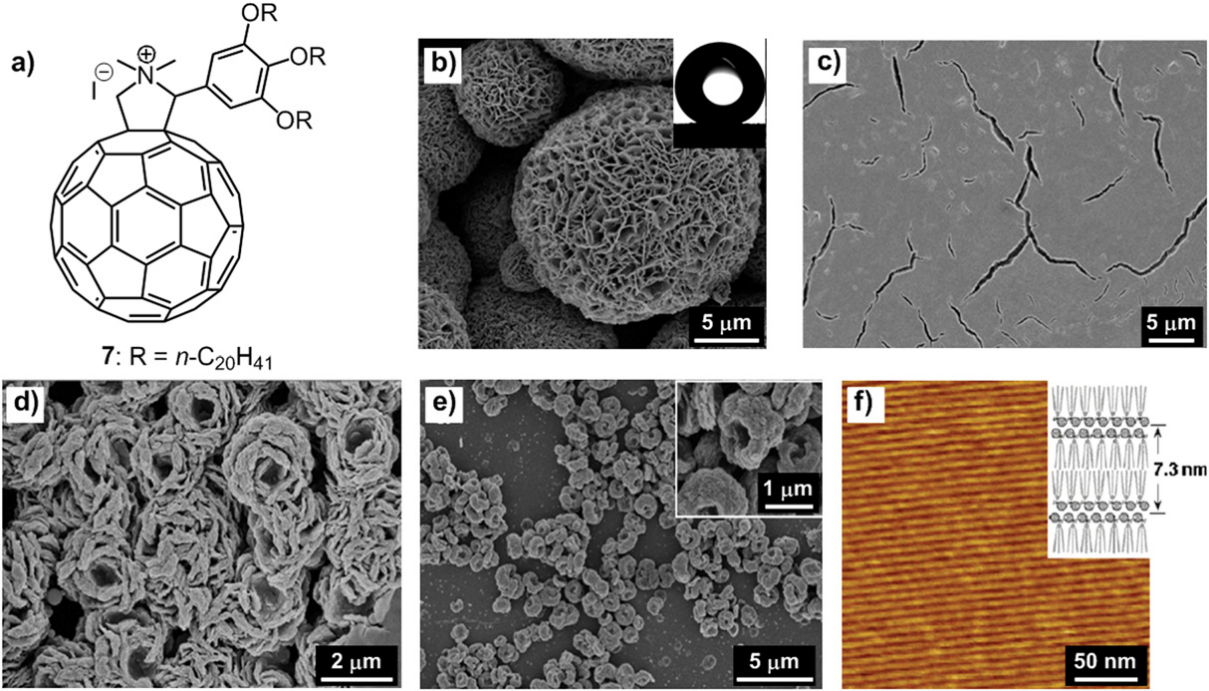
(a) Chemical structure of an ionic C_60_ derivative **7**. (b) SEM image of flake-like microparticles of **7** precipitated by slowly adding excess methanol (MeOH) to a concentrated CH_2_Cl_2_ solution of **7**; inset, a water droplet on the surface of a thin film made from the microparticles of **7**. SEM images of the self-organized structures of **7** formed on Si substrates by evaporating a 0.5 mM solution in a MeOH/CH_2_Cl_2_ mixed solvent with a MeOH volume content of 0 (c), 10% (d) and 30% (e). (f) AFM image of a film obtained by spin-coating a 10 *μ*M CH_2_Cl_2_ solution of **7** on HOPG; inset, a schematic of lamellar form of **7** on HOPG. Reprinted with permission from H Li *et al* 2011 *Langmuir* **27** 7493, © 2011 American Chemical Society.

Our group has designed an ionic alkylated C_60_ derivative, **7** (figure 9(a)), which exhibited multiple morphologies with different processing methods [59]. Through liquid-liquid interfacial precipitation with the addition of a poor solvent, methanol (MeOH), on the top of a concentrated dichloromethane (CH_2_Cl_2_) solution of **7**, self-organized flake-like microparticles with high roughness were produced (figure 9(b)). However, through drop-casting of a stock solution of **7** with CH_2_Cl_2_, CH_2_Cl_2_/MeOH = 9:1 and CH_2_Cl_2_/MeOH < 7:3 as solvents on a Si substrate, film with some cracks (figure 9(c)), closely packed flower-like objects (figure 9(d)) and doughnut-shaped micro-objects (figure 9(e)) with rough surfaces were generated, respectively. On the other hand, the diluted CH_2_Cl_2_ solution of **7** (10 *μ*M), once spin-coated on HOPG, could form perfectly straight C_60_ nanowires in which the length of nanowires exceeded 1 *μ*m (figure 9(f)).

Such polymorphic phenomenon was benefited by the introduction of an ionic unit (pyrrolidinium iodide) on the molecule, which induced electrostatic interactions to the assemblies. Consequently, the multiple *π*–*π*, vdW and electrostatic interactions drove the formation of a variety of polymorphic self-assembled structures either from the solution or on substrates. Even with an additional ionic part, a thin film of the flake-like microparticles exhibited high water repellency with a static water contact angle of 140 ± 3° (figure 9(b) inset), which was viable for the development of anti-wetting materials. In addition, the nanowires formed here, stabilized by the formation of salt bridges based on the electrostatic interactions, were the longest class of 1D C_60_ self-assemblies (>1 *μ*m), which could be promising structures toward electronic device applications.

#### 2.5.2. Hydrogen bonding

**Figure 10.**
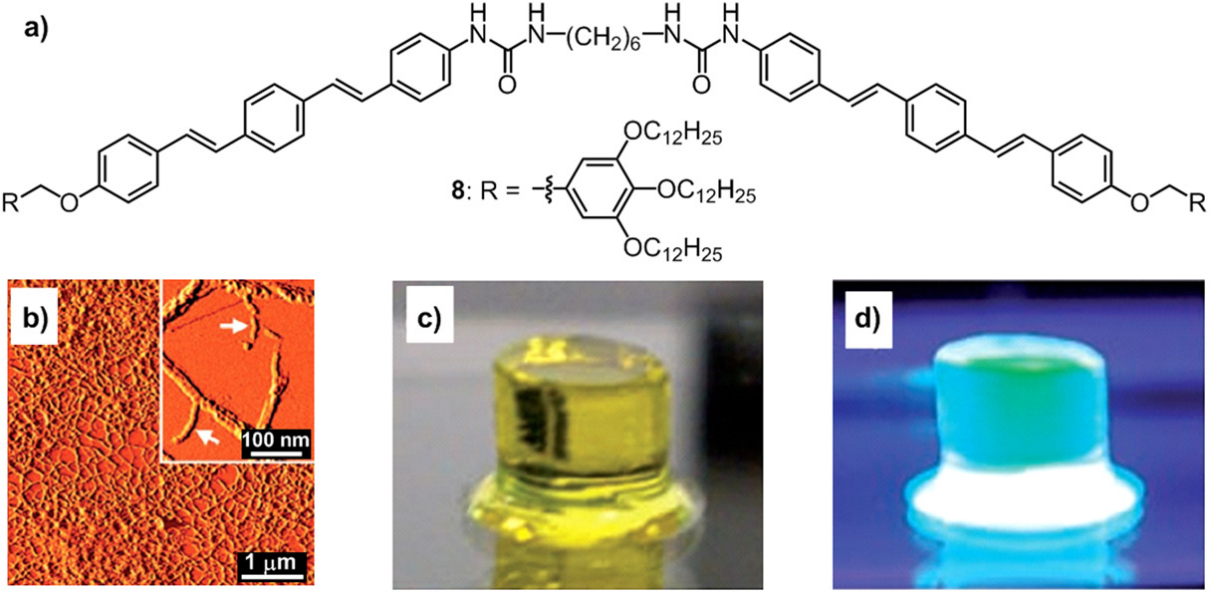
(a) Chemical structure of an OPV-functionalized bisurea **8**. (b) AFM phase images of **8** spin-coated on HOPG from a methylcyclohexane dispersion; inset shows a high-resolution image. Photographs of the self-supporting decane gel of **8** deposited on the glass substrate taken under visible light (c) and 365 nm UV light (d). Reprinted with permission from S Yagai *et al* 2008 *Chem. Eur. J.* **14** 5246, © 2008 John Wiley & Sons.

The group of Yagai synthesized a series of alkylated *π* molecules attached by hydrogen bonding units, which gave rise to numerous functional optoelectronic materials [60–62]. For example, consider an oligo(*p*-phenylene vinylene)- (OPV) functionalized bisurea, **8** (figure 10(a)) [60], as a consequence of the cooperative *π*–*π* interactions of OPV units, vdW interactions of the alkyl chains and hydrogen bonding from the urea units, the molecule possessed a very high supramolecular polymerization ability and formed noncovalent polymers with intertwined fibrous structures by simply spin-coating it in a methylcyclohexane solution on a HOPG substrate (figure 10(b)). To compare, in various organic solvents, such as *n*-decane, CH_2_Cl_2_ and THF, compound **8** showed a tendency to gelate, giving rise to fluorescent gels (figures 10(c)–(d)). Such a strong aggregation ability of **8** in a solution is significant for its applications as solution-incorporated soft materials.

#### 2.5.3. Hydrophilic interaction

**Figure 11.**
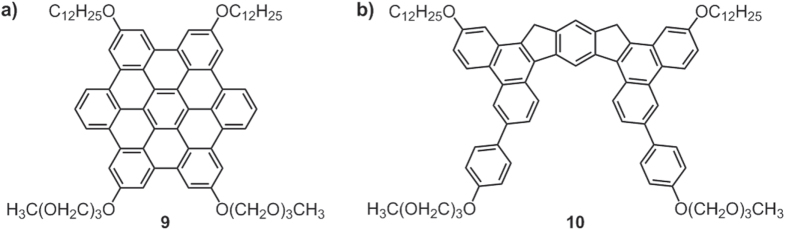
Chemical structures of a HBC derivative **9** (a) and a benzodithiophenen derivative **10** (b), both bearing hydrophobic *n*-dodecyl and hydrophilic TEG chains.

The Aida group reported a number of hexa-*peri*-hexabenzocoronene (HBC) derivatives substituted by both hydrophobic alkyl chains and hydrophilic triethylene glycol (TEG) chains [63, 64]. These molecules, take **9** (figure 11(a)) as an example, self-assembled into well-defined 1D nanotubes stabilized by the *π*–*π* interactions of the HBC moieties, vdW interactions of the alkyl chains and hydrophilic interactions governed by the TEG chains. The nanotube was shown to be redox active and had an electrical conductivity of 2.5 M*Ω* upon oxidation, which was comparable to that of an inorganic semiconductor nanotube based on gallium nitride [65].

With the same designing strategy, the Pei group reported a butterfly-shaped benzodithiophenen derivative **10** (figure 10(b)) which self-organized into free-standing bilayer films in a solution [66]. The films, bearing both hydrophobic alkyl and hydrophilic TEG chains, could be facilely transferred onto a substrate for direct device fabrication and were employed as the active layer of organic field-effect transistors (OFETs), with the highest mobility being 0.02 cm^2^ V^−1^ s^−1^. Such substrate-independent free-standing films would be promising for producing electro-active materials with large-area deposition and solution processability.

Within solvent systems, the intrinsic balance of *π*–*π* and vdW interactions and the influence of solvent polarity and temperature, as well as the chain-substrate interaction and other interactions induced by further molecular modification, could render the linear alkyl chain-substituted *π*-molecules assembled into diverse 1D, 2D and 3D structures. These nano/micro architectures have turned out to be widely applicable to fabricate anti-wetting surfaces, structural templates and various optoelectronics.

## 3. Liquid crystals of alkyl chain-attached *π* molecules

Taking advantage of their efficient carrier injection from the electrode and their carrier transporting ability, as well as their softness of the state, liquid crystals (LCs) containing *π*-conjugated units are of particular interest for flexible optoelectronic applications [67].

### 3.1. Linear alkyl chain-substitution-induced thermotropic liquid crystals

**Figure 12.**
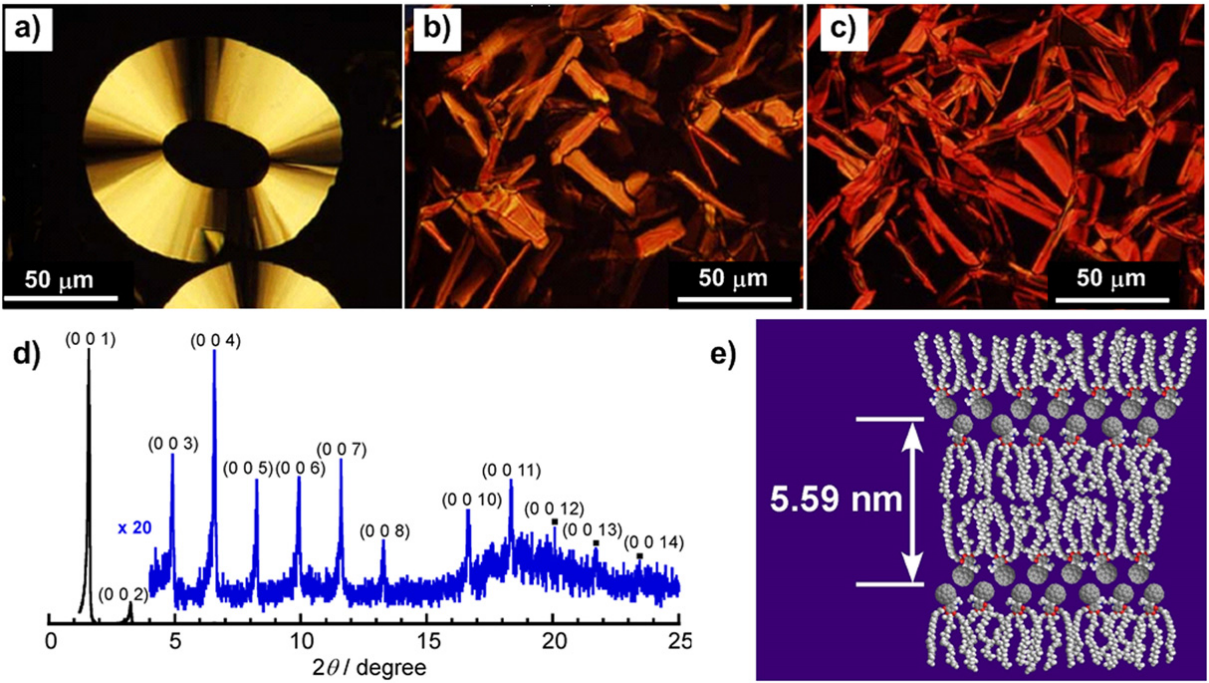
Polarized optical microscopy images of **3a** at 190 °C (a), **1a** at 202 °C (b) and **3b** at 200 °C (c) upon cooling from the isotropic state. (d) XRD patterns of **3a** at 185 °C. Reprinted with permission from T Nakanishi *et al* 2008 *J. Am. Chem. Soc.* **130** 9236, © 2008 American Chemical Society. (e) Proposed lamellar organization of **3a** (redrawn from [70]).

In spite of the promising semiconducting properties of C_60_, C_60_-containing thermotropic LCs are seldom reported, not to mention their low carrier mobility due to their moderately ordered structure and low content of C_60_ in the mesophase [68]. In view of the high C_60_ content of our alkylated-C_60,_ based on the compact molecular design, the thermal properties of the above-mentioned C_60_ derivatives were investigated. **1a**, **3a** and **3b** exhibited thermotropic polymorphism and showed unprecedentedly advantageous carrier mobility in their thermotropic mesophase [69]. For all three compounds, two endothermic peaks corresponding to crystalline-to-mesomorphic and mesomorphic-to-isotropic phase transitions were observed from differential scanning calorimetry (DSC) analysis upon heating of each sample. The thermotropic mesophase of **3a** formed in a temperature range from 62 °C to 193 °C, within which an optical texture exhibiting birefringence and fluid nature was observed under polarized optical microscopy (POM) (figure 12(a)). **1a** and **3b** showed similar LC characteristics in temperature ranges from 33 °C to 223 °C (figure 12(b)) and from 44 °C to 226 °C (figure 12(c)), respectively. Significantly, the mesophase of **3a** was able to retain the electrochemical and photoconductive properties of pristine C_60_, featuring both reversible electrochemistry (*E*_red,1_ = −0.70 V and *E*_red,2_ = −0.87 V versus Ag/AgCl) above the crystalline-to-liquid-crystalline transition temperature (62 °C) and relatively high electron mobility of ∼3 × 10^−3^ cm^2^ V^−1^ s^−1^ at 120 °C, evaluated by a conventional time-of-flight (TOF) set-up.

The high photoconductive properties of these liquid crystalline materials are attributed not only to the high content of C_60_ (up to around 50%) but also to a suitable and dense C_60_ arrangement. As mentioned above, the C_60_ moieties and the alkyl chains act as amphiphiles in an organic solvent. The high immiscibility of the two components could significantly facilitate the segregation of C_60_ microphases into layers even under solvent-free conditions, which would naturally promote the formation of desirable molecular building blocks for liquid-crystalline organization. The densely packed C_60_ structures were confirmed by x-ray diffraction (XRD) analysis (figure 12(d)). Take **3a** as an example: at 185 °C, its XRD pattern shows a strong peak at 2*θ* = 1.58° assigned to (0 0 1), *d* spacing = 5.59 nm, with a number of high-order diffraction peaks up to (0 0 14), illustrating a long-range ordered lamellar mesophase with interdigitation of only C_60_ units (figure 12(e)) [70].

With satisfying optoelectronic properties guaranteed by a highly ordered structure and high content of C_60_ in the mesophase, such C_60_-containing LCs can be employed as promising soft materials for photovoltaic applications.

### 3.2. Branched alkyl chain-substitution-induced thermotropic liquid crystals

Some branched alkyl chain-substituted *π* molecules were also found to form thermotropic liquid crystals and showed more promising properties for optoelectronic applications than those of linear ones.

**Figure 13.**
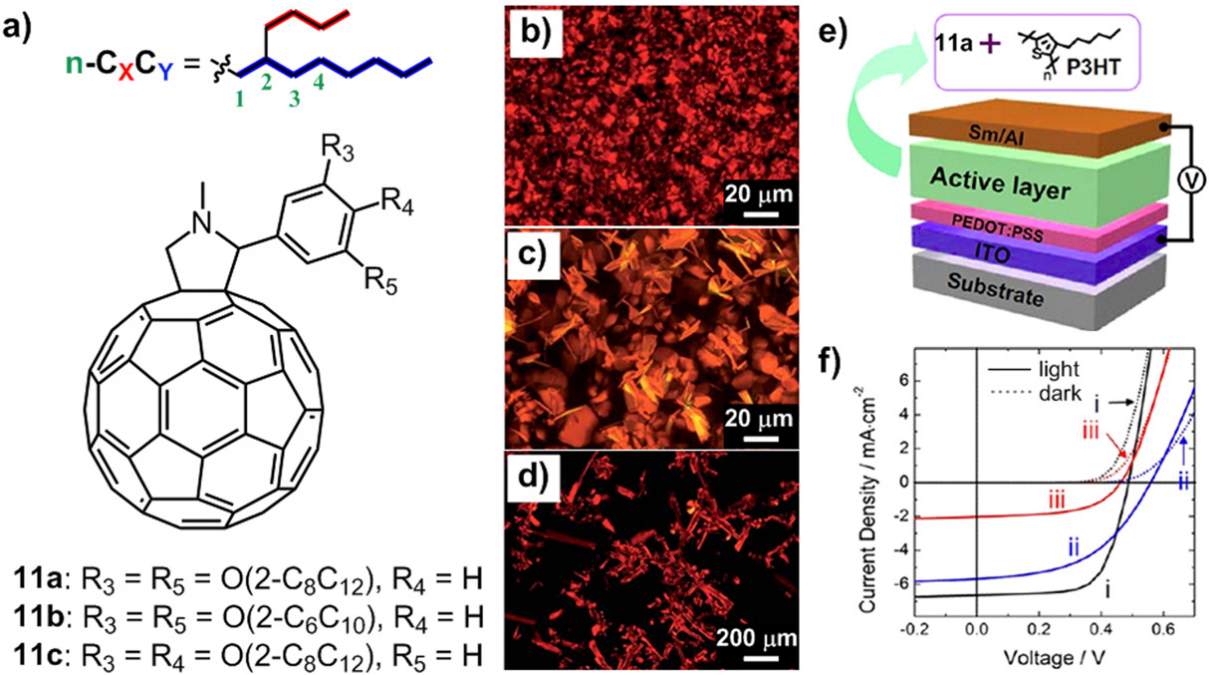
(a) Chemical structures of C_60_ derivatives **11a**-**11c** containing branched alkyl chains. POM images of **11a** (b), **11b** (c) and **11c** (d) taken in their mesophases. (e) Schematic of the BHJ cell. (f) *J*(V) curves of binary mixtures of PCBM/P3HT (curve i), **11a**/P3HT (curve ii) and **3a**/P3HT (curve iii), respectively. Reprinted from [71].

Figure 13(a) shows three C_60_ derivatives **11a**-**11c** substituted by branched alkyl chains, all of which exhibited a thermotropic smectic phase observed by POM (figures 13(b)–(d)) [71]. Compound **11a** with branched 2-octyldodecyl (2-C_8_C_12_) chains had a mesophase to isotropic phase transition at 84 °C, which was much lower than that of compound **3a** (193 °C) attached by linear eicosyl (*n*-C_20_H_41_) chains, indicating the better softening effect and lower crystalline tendency of branched chains. With shorter branched chains, compound **11b** (2-C_6_C_10_) exhibited a mesophase-to-isotropic phase transition at 148 °C, which is 64 °C higher than that of **11a**. In sharp contrast, with linear chains, molecule **1a** (*n*-C_16_H_33_) only showed a 30 °C increase of such a phase transition compared with **3a**, proving the stronger ability of branched chains to regulate the thermotropic behavior of alkylated *π* molecules. Moreover, by simply changing the substitution position of **11a** from (3-, 5-) positions to (3-, 4-) positions, the resulting compound **11c** showed a drastic increase in the phase transition temperature (196.2 °C), which was attributed to the greater vdW interaction caused by densely packed alkyl chains. As a result, chain length, substitution position and branched extent have all played significant roles in the thermotropic behavior of the alkylated C_60_ derivatives.

With the liquid-crystalline phase extended from 84 °C to room temperature, compound **11a** was selected to blend with poly(3-hexylthiophene) (P3HT) for the fabrication of bulk heterojunction (BHJ) organic solar cells (figure 13(e)). Notably, **11a**/P3HT exhibited a power conversion efficiency (PCE) of ∼1.6 (figure 13(f), curve ii), which was comparable to that of [6,6]-phenyl-C_61_-butyric acid methyl ester (PCBM)/P3HT (figure 13(f), curve i) in our cell set-up. On the contrary, **3a**/P3HT showed a much lower PCE of ∼0.5 (figure 13(f), curve iii). The dramatically enhanced PCE value of **11a**/P3HT compared with that of **3a**/P3HT was ascribed to the lowering of crystallinity and facilitating of charge transportation caused by the branched alkyl chains. In other words, too high of a crystallinity in the solar cell caused defects and reduced the performance. Therefore, ordering whilst retaining certain softness (flexibility) would play a very important role in such optoelectronic device applications.

Discotic thermotropic LCs are well known for their extensive applications in various organic devices, such as OFET [72–74] and photovoltaic cells [75], depending upon their orientation on the substrate. All these applications are derived from their fabulous ability to conduct charges on the basis of their unique molecular self-organization behaviors. In discotic LCs, the molecules stack on top of one another into columns and consequently resulted in a regular lattice, which greatly facilitates the charge carrier mobility along the 1D assembly [76–78]. A promising candidate for developing discotic LCs is HBC, which has a large aromatic core that allows one of the highest values of intrinsic charge carrier mobility for mesogenes to be obtained [79–81].

**Figure 14.**
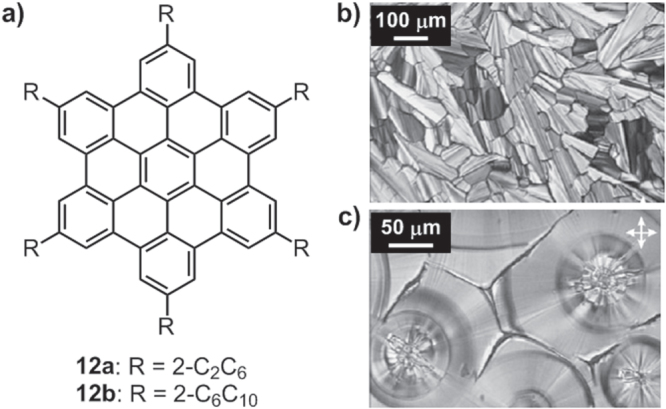
(a) Chemical structures of HBC derivatives **12a**–**12b** containing branched alkyl chains. POM images of **12a** (b) and **12b** (c) obtained after cooling from the isotropic state. Reprinted with permission from M Kastler *et al* 2006 *Adv. Mater*. **18** 2255, © 2006 John Wiley & Sons .

Müllen and his coworkers have reported a number of thermotropic LCs based on HBC derivatives substituted with both linear [73, 82] and branched alkyl chains [83, 84] (figure 14). All the linear alkyl chain-substituted HBC derivatives, together with the short branched alkyl chain-equipped HBC compound **12a**, exhibited a crystalline-to-LC phase transition around 100 °C and a LC-to-isotropic phase transition around 420 °C. In dramatic contrast, the long-branched alkyl chain-modified HBC derivative, **12b**, displayed much lower phase transitions with a crystalline-to-LC phase transition at 17 °C and LC-to-isotropic phase transition at 97 °C, which further confirmed the more profound softening effect of branched chains and their stronger ability to regulate and adjust the thermotropic behavior. At room temperature, the charge carrier mobility for **12b** was determined to be 0.73 cm^2^ V^−1^ s^−1^, representing the highest value measured for a noncrystalline discotic LC material.

The relative softness and highly ordered phases make these alkylated-*π*-molecule-constituting LCs promising materials for fabrication into flexible optoelectronic devices. The thermotropic behaviors of these LC systems depend not only on the alkyl chain length and substitution positions but also on the branching. As illustrated, branched alkyl chains possess a better softening effect as well as more pronounced influence on the self-organization and carrier mobility of these alkylated-*π* LC systems.

## 4. Solvent-free liquid of alkyl chain-attached *π* molecules

The above-described thermotropic liquid crystals were derived from linear alkyl chain-substituted C _60_ derivatives **1**, **4** and **5** where the alkyl chains resided on the phenyl unit at (3,4,5-) or (3,4-) positions. In contrast to these LCs, nonvolatile, solvent-free room-temperature liquid C_60_ derivatives were discovered serendipitously by attaching the 2,4,6-tris(alkyloxy)phenyl group to a C_60_ derivative where the alkyl chains were of a linear type [85]. This finding further confirmed the extraordinary impact of a chain substitution pattern on the intermolecular interactions, as depicted in part 2.3. These liquid C_60_s, with solvent-free device processability, nonvolatility, tunable optoelectronic functions, high density of optically or electronically active *π*-conjugated moieties and the ability to blend with other organic or inorganic dopants, have attracted remarkable attention and were quickly extended to various functional alkylated-*π* systems with diverse *π* units substituted by both linear and branched alkyl chains.

### 4.1. Linear alkyl chain-substitution-induced solvent-free liquids

**Figure 15.**
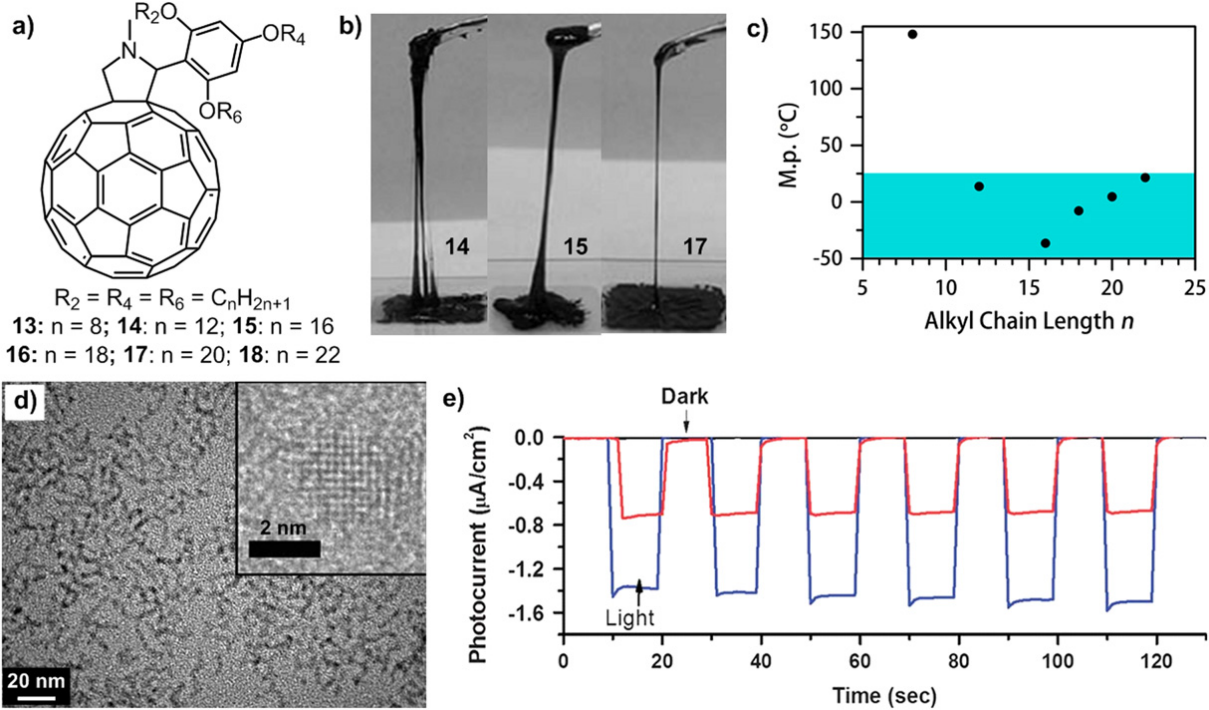
(a) Chemical structures of C_60_ derivatives **13**–**18** containing linear alkyl chains. (b) Photographs of room-temperature C_60_ liquids **14**, **15** and **17**. Reprinted with permission from T Michinobu *et al* 2006 *J. Am. Chem. Soc*. **128** 10384, © 2006 American Chemical Society. (c) Relationship between the melting point and alkyl chain length of C_60_ derivatives **13**–**18**, redrawn from [86]. (d) TEM image of the CdSe NCs/**17** composite; inset, high-resolution TEM image of a CdSe NC. (e) Photoelectrochemical activity of solvent-free CdSe NCs/**17** composite films on glass coated with fluorine-doped tin oxide containing **17** alone (black), 25 wt% CdSe NCs (blue) and CdSe NCs alone (red) under blue light (at 480 nm) illumination. Reprinted from [87].

During the investigation of alkylated-C_60_ derivatives, our group found that compounds **14**, **15** and **17** substituted with the 2,4,6-tris(alkyloxy)phenyl group (figure 15(a)) exhibited a liquid phase at room temperature with melting points of 13.7, −36.5 and 4.5 °C, respectively (figure 15(b)) [85]. The formation of such a room-temperature liquid state could be attributed to the 2,4,6-substitution pattern, which disturbed the *π*–*π* interactions of the C_60_ units based on the independent spreading of the three chains acting as an effective steric stabilizer of individual C_60_ moieties.

Interestingly, the lower-viscous liquid C_60_s were achieved through attachment of the longer alkyl chains due to the further weakening of C_60_-C_60_ interactions. This viscosity trend was confirmed by a detailed study of the impact of the alkyl chain length on the rheological behavior of **14**–**18** [86]. Notably, in addition to viscosity, the melting points of these compounds were also strongly influenced by alkyl chain lengths (figure 15(c)). With short alkyl chains (**13**: *n*-C_8_H_17_), the dominant intermolecular *π*–*π* interactions of adjacent C_60_ units would lead to a melting point higher than room temperature (147–148 °C). However, with extremely long alkyl chains (**18**: *n*-C_22_H_45_), the vdW interactions of alkyl chains became predominant and led to increased melting point (21.5 °C) compared with **14**–**17**. As a consequence, only with medium alkyl chain lengths could the two interactions balance to generate a liquid phase with the melting point under room temperature (i.e. 25 °C).

Importantly, even in the liquid state, the redox properties and the high hole mobility (∼3 × 10^−2^ cm^2^ V^−1^ s^−1^ at 20 °C measured by a TOF technique) of the C_60_ unit were retained, making these liquid molecules promising for developing electronic materials. Moreover, these highly fluidic liquids can act as a matrix for other optoelectronic-active dopants, resulting in various composite materials with multiple functions. For example, cadmium selenide (CdSe) nanocrystals (NCs) with tunable optical properties were successfully embedded in liquid **17** [87]. Rather than forming macroscopic aggregation, the NCs tended to organize in relatively short (∼10 nm) serpentine structures (figure 15(d)), illustrating the high dispersity of CdSe NCs in liquid **17**. The favorable electronic band alignment between the NCs and the **17** matrix enabled inter-phase charge transfer, which induced a dramatic increase in the cathodic photocurrent under light illumination observed for a film of composite CdSe NCs/**17** compared to the film of pure **17** or pure CdSe NCs (figure 15(e)). This result made the composite material an excellent candidate for application in photo-sensitized photovoltaic devices.

**Figure 16.**
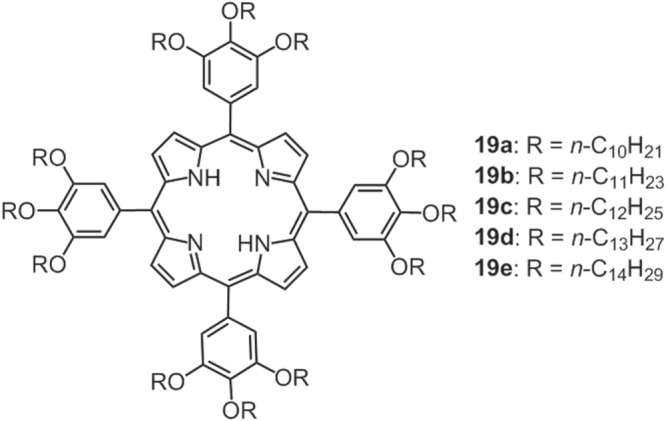
Chemical structures of liquid porphyrins **19a**–**19e** containing linear alkyl chains.

The groups of Nowak-Król [88] and Maruyama [89] have independently reported a series of room-temperature liquid porphyrins, **19a**–**19e**, by introducing long linear alkyl chains to 5,10,15,20-tetraphenylporphyrin (figure 16). The thermal properties of the compounds were very sensitive to the alkyl chain lengths, with the melting point increasing proportionally to the increase of the chain length. However, a further increase of the chain length (*n*-C_15_H_31_ [89]) would increase the melting point above room temperature, a behavior similar to that of liquid C_60_ derivatives [86].

Compound **19c** was employed as a suitable dispersion media for C_60_ and/or carbon nanotubes based on the intermolecular donor-acceptor and/or *π*–*π* interaction, which were confirmed by spectroscopic changes. Such dispersal may create new optical and electrical functionalities.

### 4.2. Branched alkyl chain-substitution-induced solvent-free liquids

As mentioned in part 3.2, branched alkyl chains possess lower crystalline tendency and a better softening ability as well as a more pronounced effect on reducing the molecular melting point than linear ones. Therefore, branched alkyl chains can be better candidates for constructing room-temperature liquid molecules containing a functional *π* unit. Accordingly, a series of room-temperature liquid materials by attaching branched alkyl chains to various *π* molecules have been reported by our group and other research groups.

**Figure 17.**
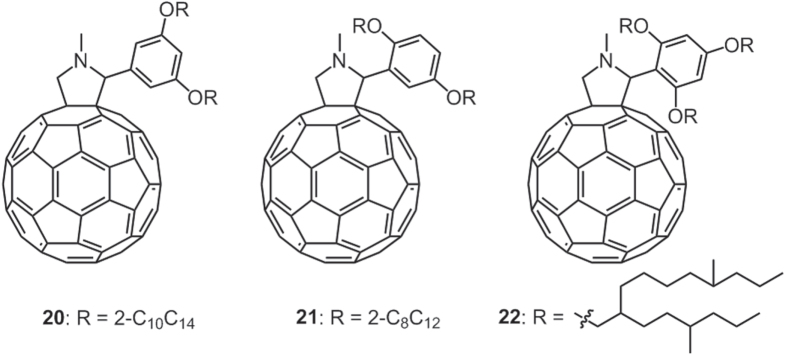
Chemical structures of C_60_ liquids **20**–**22** containing branched alkyl chains.

Our group has synthesized a number of liquid C_60_ derivatives **20**–**22** attached by either swallow-tail branched alkyl chains (**20**–**21**) or hyperbranched alkyl chains (**22**) (figure 17) [71]. Compared with the linear alkyl chain-substituted C_60_ derivatives **14**–**18**, which required a 2,4,6-substitution pattern to reach a room-temperature liquid state, both **20** and **21** needed only two branched chains to generate room-temperature liquids. More significantly, **20** and **21** exhibited not only lower melting points (<−120 °C) but also less viscosity than **14**–**18**. This was because in the case of branched chains, both the vdW interaction of the chains and the *π*–*π* interaction among the C_60_ moieties were strikingly suppressed. Interestingly, the viscosity of **21** (∼260 Pa · s) turned out to be much lower than that of **20** (∼1500 Pa · s) even though **21** had shorter branched alkyl chains. This finding emphasized the importance of substitution at the 2-position on the phenyl unit to produce less viscous C_60_ liquids.

However, a further increase of the branching extent (**22**) would lead to both an increased melting point (12 °C) and an enhanced viscosity (∼128 000 Pa · s) of the isotropic phase, which is even higher than the linear chain-substituted C_60_ liquids **14**–**18**. This is due to the hyperbranched-structure-induced high intra- and intermolecular friction during flowing.

Therefore, both the melting point and viscosity of alkylated C_60_ derivatives can be effectively reduced by suitable branching and proper substitution position of the alkyl chains. However, too high a branching extent would increase the melting point and viscosity again. These optoelectronically active-C_60_-containing liquids provide opportunities for constructing flexible, printable photovoltaic devices.

**Figure 18.**
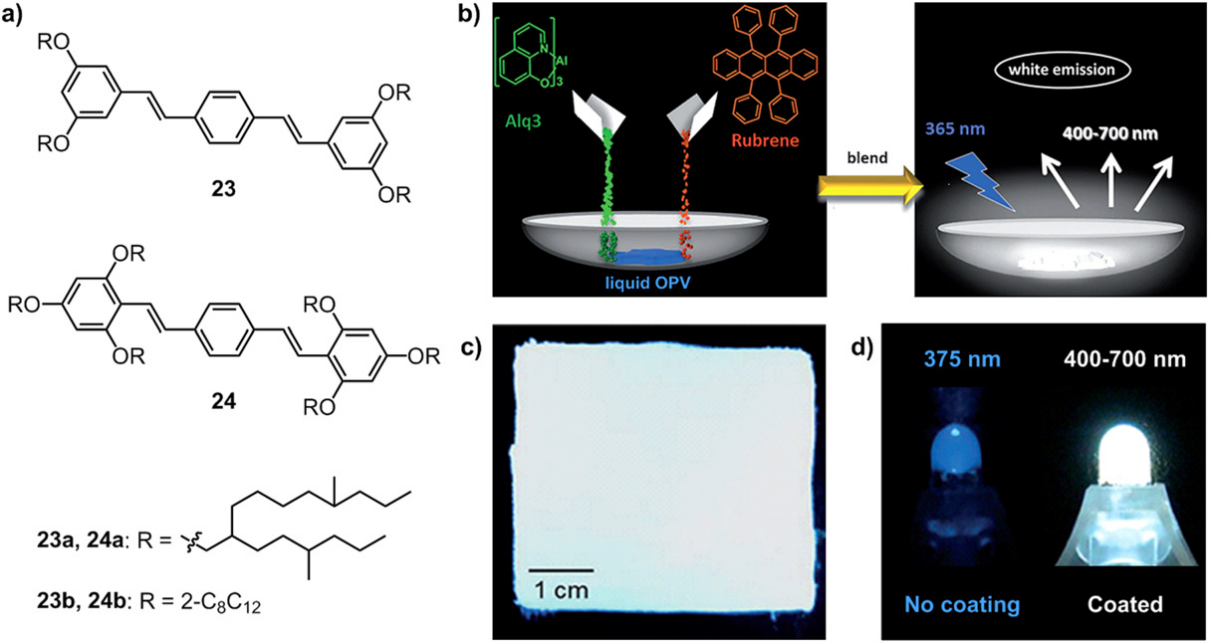
(a) Chemical structures of OPV derivatives **23**–**24** containing branched alkyl chains. (b) Schematic illustration of the preparation of a solvent-free white-emitting liquid composite using liquid OPV. (c) 5 × 5 cm^2^ area coated with the white-emitting liquid composite and exposed to UV light (365 nm). (d) Commercially available UV-LED (375 nm) before and after coating with the white-emitting liquid composite. Reprinted with permission from S S Babu *et al* 2012 *Angew. Chem. Int. Edn.* **51** 3391, © 2012 John Wiley & Sons.

Based on the designing strategy of C_60_ liquids, our group extended the *π*-conjugated C_60_ unit to an emissive *π* conjugating system and prepared OPV liquids, **23**–**24**, by substituting two different OPV units with branched alkyl chains (figure 18(a)) [90]. **23**–**24** are pale yellow fluids at room temperature with low melting points between −43 °C to −55 °C. Similar to the C_60_ liquids **20**–**22**, OPV liquids with swallow-tailed branched alkyl chains substituting on the (2,4, 6-) positions (i.e. **24**) possess much lower viscosity than OPVs with hyper-branched alkyl chains appending on the (3,5-) positions (i.e. **23**).

The UV–vis absorption and fluorescence spectroscopic properties of these liquids are almost identical to their dilute solution analogues, demonstrating efficient isolation of OPV units upon wrapping by the soft alkyl chains in the solvent-free liquid state. Taking into account the advantageous color purity and stability of white-emitter materials and the ability of molecular liquids to blend organic or inorganic dopants for producing functional hybrid materials, the blue-emitting **23b** or **24b** was employed as a matrix for doping green-emitting tris(8-hydroxyquinolinato)aluminium (Alq3) and orange-emitting 5,6,11,12-tetraphenylnaphthacnee (rubrene) to obtain white-emitting liquids (figure 18(b)). Simply blending the three components for just one minute would result in the composites exhibiting a white emission spanning from 400 to 700 nm. The composite maintained the strong emission features of OPV and had a quantum yield of over 35%, which, together with the low melting point of around −45 °C and low viscosity of 3.2 Pa · s, enabled writing or painting of the white-light-emitting material on various surfaces (figure 18(c)) such as the surface of a UV light-emitting diode (LED) (figure 18(d)).

**Figure 19.**
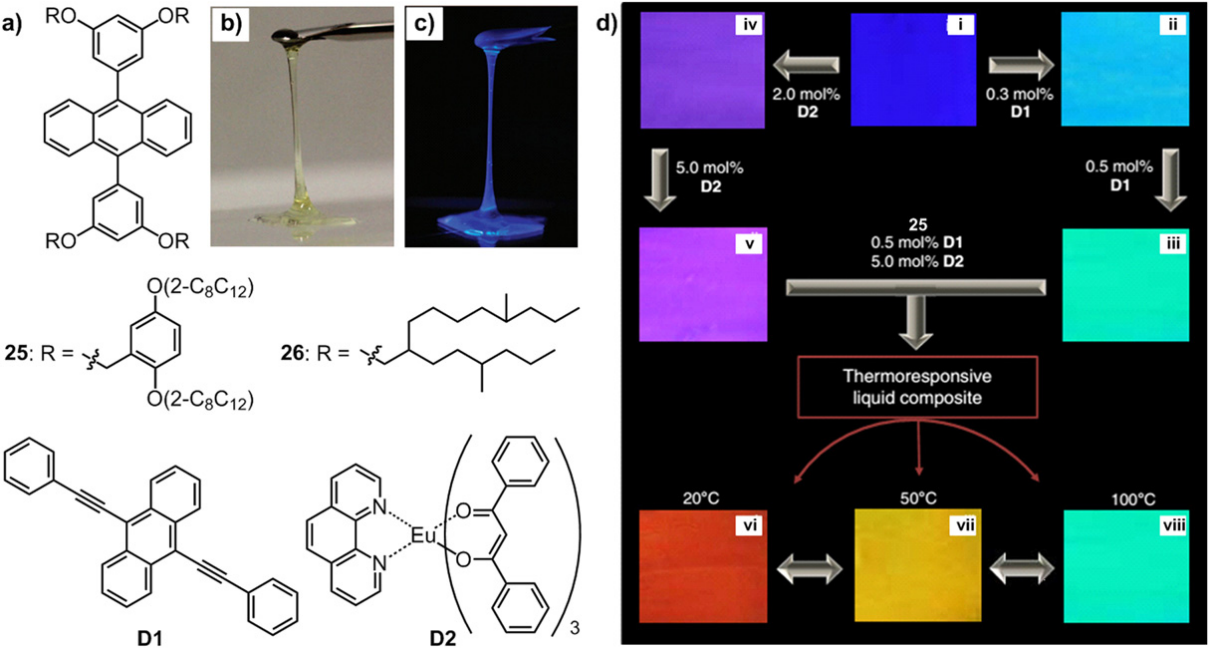
(a) Chemical structures of anthracene derivatives **25**–**26** containing branched alkyl chains and dopants **D1** and **D2**. Photographs of **26** under visible (b) and UV light (365 nm) (c). (d) Photographs of the luminescence color tunability and thermal response of the composites of **25**, **D1**and **D2**: (i) **25** alone; (ii) **25** + **D1** (0.3 mol%); (iii) **25** + **D1** (0.5 mol%); (iv) **25** + **D2** (2.0 mol%); (v) **25** + **D2** (5.0 mol%) and **25** + **D1** (0.5 mol%) + **D2** (5.0 mol%) at 20 °C (vi); 50 °C (vii) and 100 °C (viii). Adapted from [91] under a Creative Commons Attribution 3.0 Unported (CC BY) license.

Right after this achievement, our group synthesized another type of blue-emitting liquid, **25**–**26** (figure 19(a)), by attaching branched alkyl chains to anthracene-emitting units [91]. Both compounds are yellowish transparent viscous liquids under visible light (figure 19(b)) but show blue luminescence under UV light (figure 19(c)) due to the reduction of *π*–*π* interactions through soft alkyl chains substitution. Compound **25,** with eight suitable branched alkyl chains, exhibits a lower melting point and viscosity than **26**, with only four hyperbranched chains, which is highly consistent with the behaviors of C_60_ and OPV liquids.

Enveloped by the alkyl chains, the anthracene cores were effectively isolated, resulting in similar absorption and fluorescence spectra of **25**–**26** in the solvent-free state and dilute solution. In addition, such enveloping can effectively prevent oxygen attack and dimerization of the anthracene cores, resulting in remarkably improved photostability, which was confirmed by time-dependent nuclear magnetic resonance (NMR) and emission intensity upon Xe-lamp irradiation.

Upon doping **25** with 9,10-bis(phenylethynyl)anthracene (**D1**) and tris(1,3-diphenyl-1,3-propanedionato)-(1,10-phenanthroline)europium(III) (**D2**), its luminescent color can be freely tuned based on an energy transfer mechanism, according to which **25** acts as a Förster resonance energy transfer (FRET) donor for both **D1** and **D2**. As shown in figure 19(d), with the increasing mol% of **D1**, the blue emission (i) of **25** changed to cyan (ii, 0.3 mol%) and green (iii, 0.5 mol%), while in the case of **D2**, the emission color of **25** changed to violet (iv, 2.0 mol%) and purple (v, 5.0 mol%). Moreover, by blending liquid **25** with both **D1** (0.5 mol%) and **D2** (5.0 mol%), a red-emitting composite (vi, 20 °C) was obtained. Upon increasing the temperature, the red emission changed to yellow (vii, 50 °C) and emerald green (viii, 100 °C) by virtue of the well-known temperature-dependent emission of **D2**, allowing the composite to be used as a color-indicating thermometer. Such facile luminescent color-tuning spanning almost the whole visible range provides technological potential for a continuous active layer in flexible materials.

**Figure 20.**
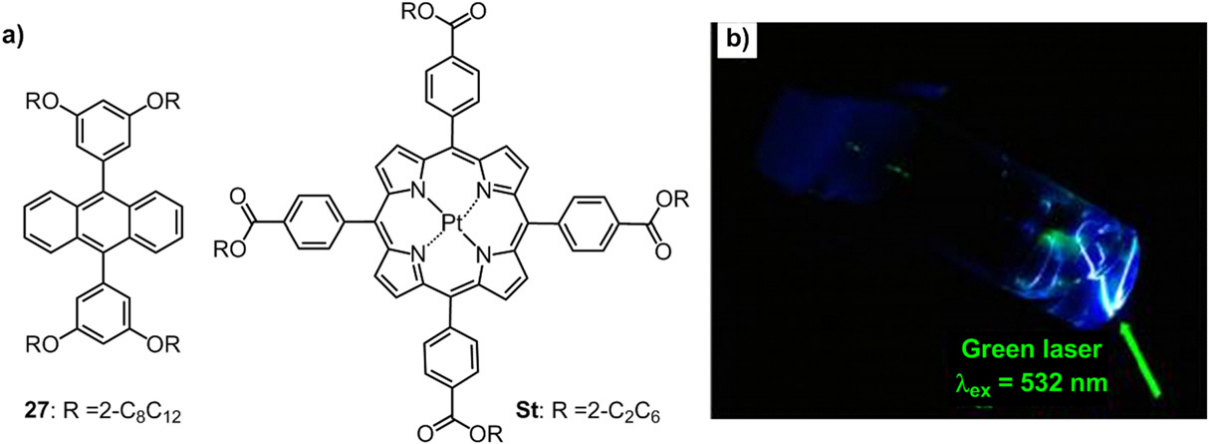
(a) Chemical structures of an anthracene derivative **27** and a Pt(II) porphyrin photosensitizer **St**. (b) Photograph of the doped liquid (**St**/**27** = 0.01 mol%) exposed to a 532 nm laser. Reprinted with permission from P Duan *et al* 2013 *J. Am. Chem. Soc.* **135** 19056, © 2013 American Chemical Society .

Inspired by our research on photostable liquid luminophors, the Kimizuka group synthesized an anthracene liquid, **27** (figure 20(a)), and applied it to an upconversion (UC) luminescent system [92]. Liquid **27** can accommodate a Pt(II) porphyrin photosensitizer, **St**, resulting in UC properties upon light irradiation with a 532 nm green laser (figure 20(b)). Compared with a traditional UC system, which was functionalized in an organic solvent and suffered emission quenching by molecular oxygen, this new UC system exhibited strong emission features, with a high quantum yield of 28% free from the oxygen effect due to the oxygen-impermeable solvent-free liquid system.

**Figure 21.**
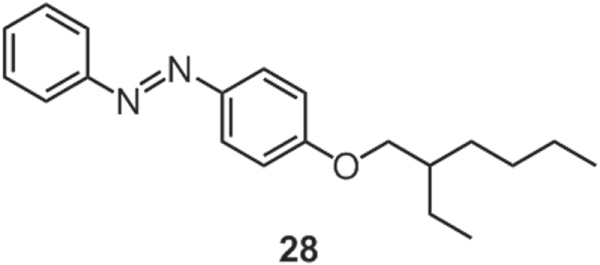
Chemical structure of an azobenzene derivative **28**.

Our group synthesized a liquid azobenzene through substitution of a branched 2-octyldodecyl (2-C_8_C_12_) chain (*vide infra*) [17]. Quite recently, Masutani *et al* [93] reported a similar liquid azobenzene **28** (figure 21) attached with a 2-ethylhexyl (2-C_2_C_6_) chain and explored its application as solar thermal fuel, a material in which light energy could be converted to chemical bond energy and concequently discharged as heat upon external stimuli. As a popular molecular photo-switch, trans-azobenzene is a hot candidate for solar thermal fuels because the photon energy can be stored in the photochemical generated cis isomer in the form of molecular strain energy and can be released as heat through cis-to-trans thermal isomerization. Conventional photoisomerization of azobenzene always occurs in a dilute solution, resulting in a remarkable decrease of the total volumetric energy density. Nevertheless, the azobenzene liquid, **28**, overcame this problem by facile photoisomerization even in a neat state, with a trans-to-cis rate comparable to those observed in a solution state. With such an excellent performance, liquid **28** could be promising for solar thermal storages.

**Figure 22.**
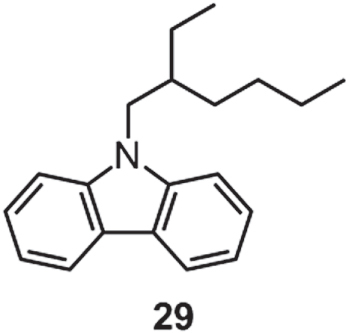
Chemical structure of a carbazole liquid **29**.

Through attachment of a 2-C_2_C_6_ chain to the nitrogen atom, a room-temperature carbazole liquid **29** (figure 22) was obtained, which was employed as a ‘solvent’ in the ellipsometry measurement for the determination of electric-field-induced birefringence in photorefractive polymer composites [94]. Later, the Wada group investigated the hole mobility of **29**, which was determined to be 4 × 10^−6^ cm^2^ V^−1^ s^−1^ by TOF experiment [95]. More recently, the Adachi group applied **29** as a liquid-emitting layer in organic light-emitting diodes (OLED) [96]. Such liquid emitters, although suffering inevitable long-term degradation in an OLED, could be facilely replaced by a flow of fresh ones, which effectively solved the problem of OLED degradation resulting from the decomposition of organic materials. Moreover, even with significant device bending, the detachment between the liquid-emitting layer and electrodes could be completely avoided, allowing the realization of flexible displays. Significantly, by doping a small amount of electrolytes into the liquid-emitting layer to decrease the driving voltage and by inserting a TiO_2_ hole-blocking layer to improve the carrier balance, the liquid OLED exhibited a maximum external electroluminescence (EL) quantum yield of 0.31 ± 0.07% and a maximum luminance of nearly 100 cd m^−2^.

Upon substitution of various *π*-conjugated molecules by alkyl chains with a proper substitution position or suitable chain branching extent, a number of room-temperature solvent-free liquids with high stability and distinctive applications were achieved. The physical liquid properties of these molecules are highly dependent on the substitution position, length and branching extent of the alkyl chains. In general, branched chains are proven to be more effective to reduce both melting point and viscosity than linear ones. However, further increasing the branching extent would increase the melting point and viscosity again. Based on this designing strategy, various optoelectronic liquids are expected to be constructed and applied to flexible-foldable device fabrications in the future by employing different functional *π*-conjugated units.

## 5. Directed assembly from solvent-free liquid

Compared with both solid self-assemblies and liquid crystals, the solvent-free liquid molecules, although possessing facile processability, suffered deficient orders due to the efficiently reduced *π*–*π* interactions of the *π* units. This shortcoming could limit their applications because high ordering of *π*-conjugated molecules is critical for optoelectronic devices for efficient exciton diffusion and electron transport. To address this issue, our group established a straightforward way for rebalancing the *π*–*π* and vdW interactions of solvent-free liquid alkylated-*π* molecules by the introduction of either alkane or *π* additives. As a result, the liquid materials were directed to be various highly ordered self-assemblies upon the addition of molecular segments.

**Figure 23.**
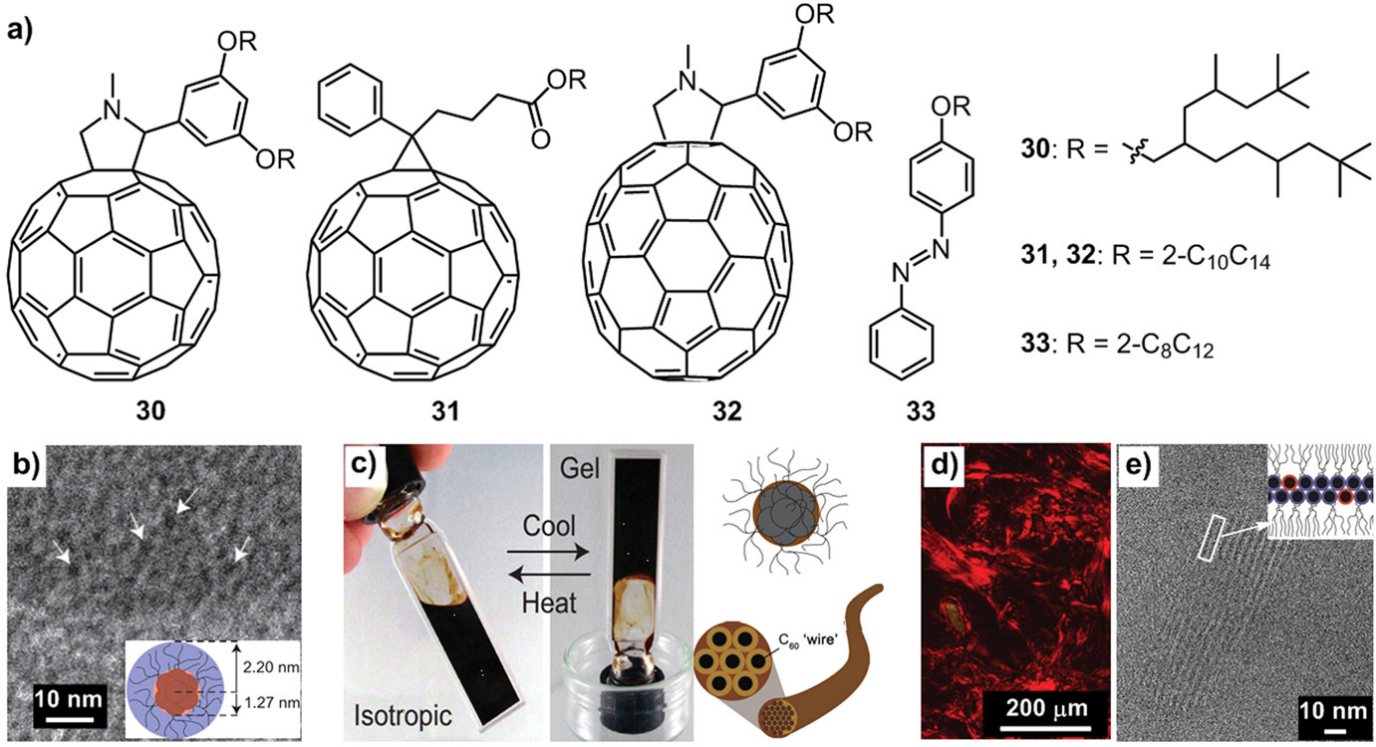
(a) Chemical structure of molecular liquids **30**–**33** containing branched alkyl chains. (b) Cryo-TEM image of micelles of **20** in *n*-decane; inset, proposed aggregate structure of **20**. (c) Left: photographs showing the isotropic and gelled states that arise on dissolving **30** in *n*-decane; right: schematic depictions of the assembled micelles in the isotropic state (up) and gel fibers in the gelled state (below). POM (d) and TEM (e) images for 1:10 molar ratios of C_60_ and **20** at room temperature; inset of (e) proposed lamellar structure. Reprinted with permission from M J Hollamby *et al* 2014 *Nat. Chem.* **6** 690, © 2014 Macmillan Publishers.

C_60_ derivatives **20** (figure 17) [71] and **30** (figure 23(a)) [17], alkylated with different branched chains, formed unstructured liquid and a disordered amorphous state at room temperature, respectively. However, upon the addition of *n*-alkanes solvents, *n*-decane for instance, the two compounds self-assembled into spherical core–shell micelles with an average diameter of 2.5 ± 0.3 nm (figure 23(b)) and into hexagonally packed gel-fibers containing insulated C_60_ nanowires with cylindrical micelles of 3.2 nm diameter (figure 23(c)), respectively.

As elucidated in part 2.1, the alkane solvents showed stronger affinity to the branched chains than to the C_60_ moieties due to the unconventional amphiphilic features of the two moieties in an organic solvent. Therefore, similar to the additive-directed assembly of conventional hydrophobic-hydrophilic surfactants in aqueous media, the introduction of alkanes would be able to direct the assembly of such alkyl-*π*-conjugated molecules in organic media. Compared with **20**, compound **30** with an increased branching number of alkyl chains suffered reduced interaction strength with the solvent molecules and weakening interference in *π*–*π* interactions between neighboring C_60_ units. As a result, compound **30** yields larger assemblies than compound **20**.

Similarly, additives with higher affinity to the C_60_ part rather than the alkyl chains were introduced; these additives were also capable of driving the assembly by strengthening the *π*–*π* interactions of the C_60_ moieties. For example, the addition of pristine C_60_ to **20** would direct the assembly into the lamellar mesophase (figures 23(d)–(e)).

In addition, the assembled materials, with a large fraction of optoelectronically active components, exhibit extremely high photoconductivities of a similar order as that for solid crystalline C_60_ derivatives, including PCBM [97, 98]. Therefore, the additive- (molecular segments) directed assembly strategy developed here creates a new rule to construct assembled molecular materials with distinctive functionality and complexity. Encouragingly, this strategy can be extended to another branched chain alkylated-C_60_ derivative **31** and other alkyl-*π*-conjugated molecules including both larger C_70_ (**32**) and smaller azobenzene (**33**) *π*-conjugated systems than C_60_.

The method developed here has opened a new gateway for facile state control of alkyl-*π* molecules, allowing us to take full advantage of the easy processability of the liquid state and the long-range order-benefiting optoelectronic properties of the additive-directed assembled liquid-crystalline mesophase. Upon controlling the occurrence, assembled structures and functions of the assembled alkyl-*π* molecules from their disordered liquid state, various optoelectronic materials can be obtained which could be used for extensive practical applications in the field of flexible electronics.

## 6. Conclusions

This review covers a diversity of alkylated-*π* molecular systems with various *π*-conjugated units attached by different types of alkyl chains through chemical modification. The corporative vdW interactions of the alkyl chains and *π*–*π* interactions of the *π*-conjugated moieties affect the physical states and applications of the corresponding molecules. We have not only clarified the relationship between the balance of the two interactions and the physical states of these alkylated-*π* molecules but also reviewed their state-dependent optoelectronic properties toward various practical applications. With these extensive investigations, a clear guidance for molecular state control through alkyl-*π* engineering is provided, which can be realized by selecting proper alkyl chain types and modulating chain substitution patterns as well as by the introduction of additives. Based on this strategy, functional alkylated-*π* molecules with expected states and applications could be constructed such as anti-wetting surfaces fabricated from solid self-assemblies, BHJ organic solar cells with high PCE constructed from thermotropic LCs and photostable, full-color tunable luminescent systems formed from solvent-free liquid fluorophores. Hopefully, the alkyl-*π* engineering method summarized here would remarkably enrich the alkylated-*π* materials toward abundant high-performance optoelectronic applications.
